# Supplementary figures and images for: Are Bangladeshi healthcare facilities prepared to provide antenatal care services? Evidence from two nationally representative surveys
Source: PLOS Glob Public Health. 2022 Jul 25;2(7):e0000164. doi: 10.1371/journal.pgph.0000164 (PMC10021659; doi:10.1371/journal.pgph.0000164)

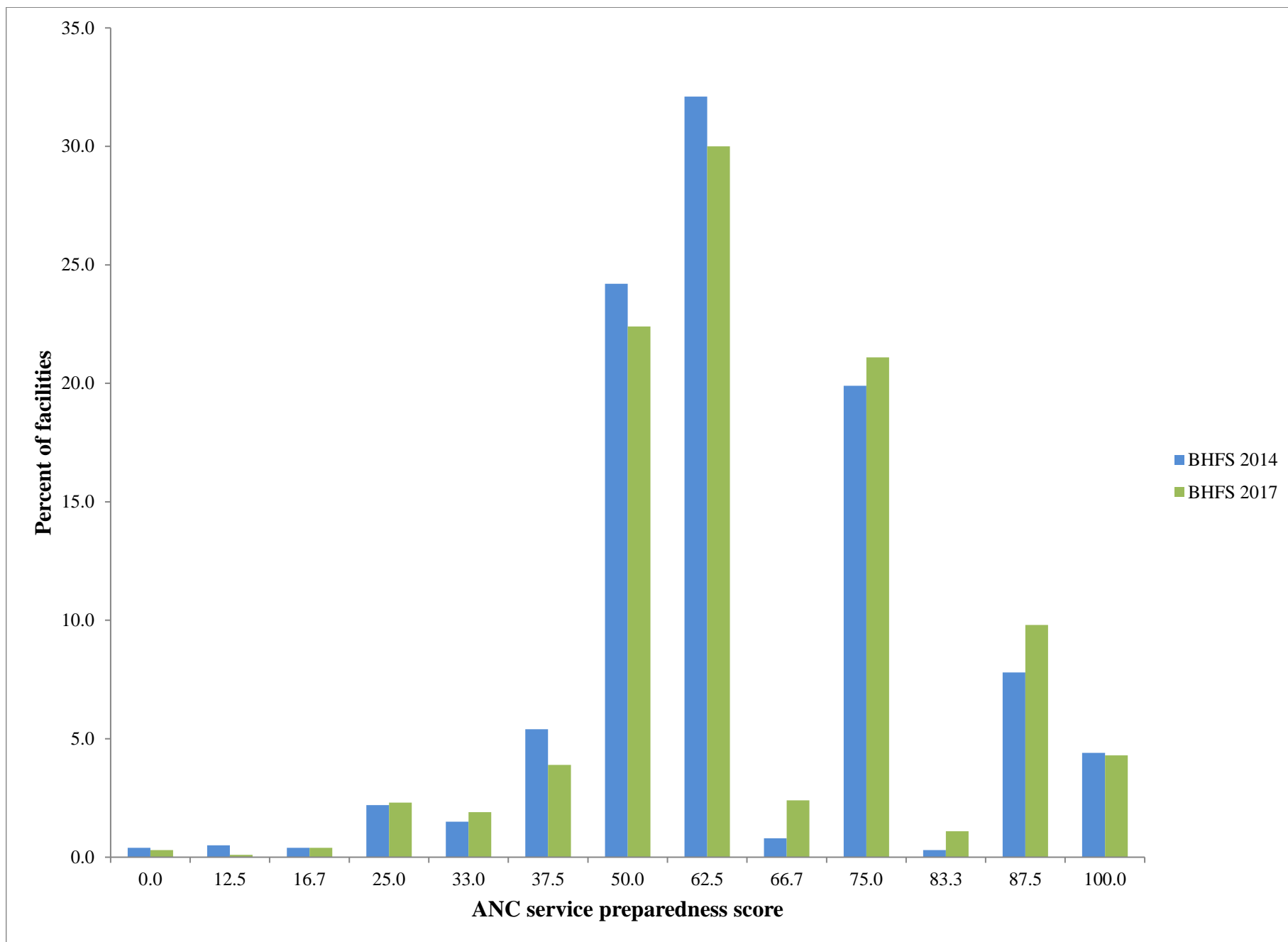

Supplement: S1 Fig — (PDF) [file pgph.0000164.s001.pdf]
