# Supplementary material for: Are Bangladeshi healthcare facilities prepared to provide antenatal care services? Evidence from two nationally representative surveys
Source: PLOS Glob Public Health. 2022 Jul 25;2(7):e0000164. doi: 10.1371/journal.pgph.0000164 (PMC10021659; doi:10.1371/journal.pgph.0000164)
Supplement: Attachment — Submitted filename: Response to reviews_PGPH-D-21-00564_R1.docx [file pgph.0000164.s002.docx]

**Reviewer #1:**

Thank you for the opportunity to review the manuscript entitled, “Are Bangladeshi health care facilities prepared to provide antenatal care services?  Evidence from two nationally representative surveys.” I appreciate the authors’ use of DHS data in 2014 and 2017 to understand the ability of health facilities to provide antenatal care to pregnant women. These sorts of analyses are important for planning maternal and women’s health services; however, I think the analysis requires some additional clarity and inputs.

Author’s response: We thank the reviewer for the thoughtful comments and suggestion.

MAJOR
1. Please note tables 1 and 2 were cut off in the submission; I could not, therefore, review them fully.

**Author’s response:** We added the tables correctly in the revised version of the manuscript.

2. The definition of readiness used in the study requires additional clarification. Of the 4 tracer indicators, and 6 individual measures that make up the “readiness score” it is used as a score and sometimes as a percentage throughout the paper. It is confusing and should be streamlined. For example, the authors report in the Methods section that that a score of 100 means the facility is completely prepared; however they report in the Results that the overall score was “low in both the survey years (4.4 and 4.3).” To be clear to the readers, please clarify which score is used, the possible range of the scores and how it should be interpreted.

**Author’s response:** Thank you for raising an important point**.** In the methods section, we mentioned that the outcome variable “ANC service preparedness” was calculated as an index score using WHO definition of service preparedness index where six indicators are considered for every facility and added up to create mean preparedness score. To get a percentage value of preparedness score, at last mean score of the four domains was multiplied by 100. Hence, this preparedness index was continuous variable which values lies between 0 and 100. For example, if a facility has a score of 100, it means that the facility is completely prepared to offer ANC service, or if a facility has a score of 50, the facility has 50% preparedness to offer the service. In the results section (page ..), we indicated that the facilities that offer ANC services, only 4.4% and 4.3% have all tracer indicators (i.e. completely prepared) to offer quality ANC in 2014 and 2017 respectively (Appendix Table 1) i.e. in 2014, 4.4% facilities scored 100 and which was 4.3% in 2017.

3. A dichotomization of “prepared or unprepared” to provide antenatal care is challenging when there are only 4 tracer indicators use in the scoring and provision of at least 50% of the indicators defines a facility as “ready.” From a clinical and implementation perspective, this is very concerning that a mere half of the 4 necessary services to be provided in antenatal care would be acceptable. In Haiti and Kenya papers the authors refer for the cutoff (reference 29 and 30), a three-level outcome was used (low, medium, and high readiness). In reference 31, a study from Tanzania, Bintabara et al required that 50% of items in each domain were required before a facility could be considered prepared. The authors of this manuscript focused on Bangladesh need to reconsider their definitions and align with the current literature or provide justification for a binary cutoff at 50%.

**Author’s response:** Similar to the Kenyan [1], Haitian [2], and a Tanzanian [3] study, we categorized the outcome variable into three categories (low, medium, and high preparedness) the distribution of preparedness scores was categorized into three equal parts based on the quintiles. The facilities having the preparedness score less than 62.5,” “between 62.5 and 66.7,” and “greater than 66.7%” were taken into account to have low, medium and high preparedness for providing ANC services, respectively.

4. As noted in comments 2 and 3, I am concerned with the application of a 50% cutoff to determine a facility as ready to provide antenatal care and how that affects the authors recommendations in the discussion. It would be helpful to reflect the impact of such low preparedness on maternal health in the Discussion.

**Author’s response:** We ignored the 50% cut off point to identify a facility as prepared to offer antenatal care. We have reanalyzed the data using new outcome variable with three categories (low, medium, and high)**.**We provided a discussion on impact of low ANC preparedness.

5. Can the authors provide more details about the choice of the “potential factors” that influenced ANC services preparedness? Several of the potential factors may be considered preparedness in and of themselves and thus appear highly correlated, even defining, the outcome; in particular, one could expect that the infection safety precaution guideline and the basic amenities score would be highly correlated or even define preparedness.

**Author’s response:** Based on the prior studies on preparedness of health facilities, the 17 potential factors were selected to identify how these are connected with the preparedness of facilities to offer ANC services [4-6]. In this study, we used a group of tracer indicators requires to be available for a health facility to be regarded as prepared to provide quality ANC services according to the WHO-Service Availability and Readiness Assessment (SARA) reference manual [7]. The outcome variable “ANC service preparedness” was calculated as an index score using WHO definition of service preparedness index where six indicators (guideline on ANC, staff trained in ANC, blood pressure apparatus, hemoglobin test, urine protein test, and iron or folic acid tablets) are considered for every facility. Since, infection safety precaution guideline and the basic amenities score are not included in the SARA reference manual as indicators of ANC service preparedness; we used these variables in this study as potential factors.

6. Please clarify in Table 4 if the multivariable survey logistic regression model included all the potential factors in a single model and thus, the table reports adjusted Odds Ratio and CI? Pleases add a footnote to the table to make this clear.

**Author’s response:** The table reports adjusted results and we cleared in the title of the table 4**.**

7. The discussion provides many good points about the correlation between potential factors and ANC preparedness. Currently, the discussion comments on every factor much like shared in the results. It would be helpful if the authors could connect the different factors to provide a summary.

**Author’s response:** We provided a summary with the different factors responsible for ANC preparedness in the discussion section.

8. In the discussion section, the paragraph focused on regional versus geographical variations is contradictory; the first sentence states there are significant regional variations, and the final sentence says there are not any significant differences. Please clarify.
**Author’s response:** The word should be regional variations. We have corrected this in the discussion.

MINOR
1. Clarity is required around authorship. On the PLoS GPH form there are three authors listed, on the title page, there are four. Zobayer Ahmed, MDS, MSS is not listed on the PLoS GPH submission paperwork. He is listed on the preprint, so I assume this was an oversight.

**Author’s response:** Yes it was an oversight. We added him on the Plos GPH**.**

2. I do note that the paper was submitted to two preprint servers but that was not noted by the authors. I believe preprints are acceptable to PLoS journals and their policies; however, I think it should be noted in the paper that this was submitted as preprint. They can be found at: <https://assets.researchsquare.com/files/rs-418861/v1/d7d15650-5781-4c60-8735-2bde6482e60c.pdf?c=1631881237> and <https://europepmc.org/article/ppr/ppr314736>

**Author’s response:**

3. As the readership of PLoS GPH is wide, it would be helpful for the authors to provide more context in the “facility type” definition. It is unclear what level each of the facilities is and how the facilities connect to each other.

**Author’s response:** We clarified the “facility type” through a flow chart. Please see page and figure 1.

4. Please provide the total number of facilities in each column in tables 2 and 3.

**Author’s response:** We provided the total number of facilities in each column in tables 2 and 3

**Reviewer #2:** This study provides an assessment of facility characteristics linked to ANC service preparedness in Bangladesh in 2014 and 2017. Overall, the study uses an excellent data source and employs the appropriate statistical methods. However, the scope of the paper is limited, and I would have appreciated a more detailed analysis on the ANC service preparedness variable and how it links (or is hypothesized to link) to better health outcomes.

Author’s response: Thank you for taking the time to provide thoughtful feedback on the manuscript.

Major Comments

- In the abstract, the authors states that a goal was “to assess whether health care facilities in Bangladesh are prepared to provide ANC service”. Currently, the analysis does not answer this question. I would recommend providing a more detailed discussion on the ANC preparedness index by either comparing the distribution of scores in Bangladesh to other countries or WHO reports on this indicator.

**Author’s response:** We thank the reviewer for pointing this out. We corrected the objective of the study in the abstract. We provided a detailed discussion of ANC preparedness of Bangladesh to other countries.

- Why did the authors choose to model preparedness as a binary variable instead of continuous or categorical? I see that other studies have chosen to dichotomize at 50%, but it seems that a lot of information is lost by doing this. The authors may want to consider at least showing the distribution of the raw scores and comparing the distribution of scores by factors using a non-parametric test (e.g. a supplemental table that contains the median and interquartile ranges for ANC preparedness by the factors – similar to Table 3).

**Author’s response:** We agree with the reviewer about the more general overarching statements in our manuscript. Similar to some prior study [1-3], based on the quintiles, the distribution of preparedness scores was categorized into three equal parts (low, medium, and high).
In table 3, we mentioned the distribution of the scores by potential factors.

Minor Comments:

- Was the information for the survey self-reported by the facility or observed by an impartial data collector? If self-reported, this should be listed as a limitation.

**Author’s response:** The information of the Bangladesh Health Facility Surveys was collected by the neutral data collectors and they interviewed the person most knowledgeable about the facility’s organization and/or the most knowledgeable provider of each service.

- Was the same sampling frame used for both surveys? In addition, what were the strata used for stratified random sampling? I realize this information is provided elsewhere, but it would be helpful to define the strata and any additional clustering.

**Author’s response:** The same sampling frame was used in both surveys. Stratification was achieved by separating the health facilities by facility type within each division.

- I want to clarify that the authors excluded facilities that did not offer ANC services. If that is the case, the language in “Selection of study samples” could be a bit clearer to reflect this.

**Author’s response:** We clarified this by changing the title into “Study samples”

- Please clarify what is meant by “managing authority”.

**Author’s response:** Managing authority means the ownership of facility.

- The authors use a cutoff of 50% for the basic amenities and infection prevention score – has this been done in prior literature? Similar to the Major Comment on the ANC preparedness, can the authors provide the distribution of this variable for their study population?

**Author’s response:** Similar to the outcome variable, the distribution of the basic amenities and infection prevention scores were classified into three equal segments (low, medium, and high) based on quintiles. We provided the distribution of these two variables for their study population in Table 3.

- Do the sampling weights account for non-response?

**Author’s response:** The BHFS weights were calculated by the DHS program and included in the final data set. The health facility weight was calculated based on the health facility selection probability, adjusted for non-response at the sampling stratum level.

- Was there any missing data in the outcome or factor variables? If so, how was this handled?

**Author’s response:** There were no missing values in the outcome variable. But there were 15 and 14 missing values in the variable “Routine quality assurance activities” in the 2014 and 2017 survey respectively. The missing values were excluded in the regression modeling.

- Does “availability of tracer indicators” mean the “% of health facilities that have information available on the indicator” OR is it the “% of health facilities that provide that service”. The text makes it seem like the former, but I am assuming the authors meant the latter. Please clarify in the text.
**Author’s response**: “Availability of tracer indicators” indicates among facilities offering antenatal care (ANC) services, the percentage having the indicators on the day of the survey.

We clarified it in the text.

- In the Univariate Analysis and Bivariate Analysis results section, the authors report results comparing 2014 and 2017, but from the Introduction and Methods sections, it seems like understanding preparedness by the “factors” is more important – and this is actually what the statistical tests are testing. I would recommend discussing the differences in preparedness within each factor, rather than focusing on changes over time.

**Author’s response:** Thanks for your valuable comments. In the discussion, we mentioned the differences in preparedness within each factor.

- Please include your Stata code for the analysis to ensure this research is reproducible.

**Author’s response:** We have uploaded codes.

1.	Agwanda, A., A. Khasakhala, and M. Kimani, *Assessment of family planning services in Kenya: Evidence from the 2004 Kenya Service Provision Assessment survey.* 2009.

2.	Wang, W., M. Winner, and C.R. Burgert-Brucker, *Limited service availability, readiness, and use of facility-based delivery care in Haiti: a study linking health facility data and population data.* Global Health: Science and Practice, 2017. **5**(2): p. 244-260.

3.	Bintabara, D., et al., *Client satisfaction with family planning services in the area of high unmet need: evidence from Tanzania Service Provision Assessment Survey, 2014-2015.* Reproductive health, 2018. **15**(1): p. 1-9.

4.	Bintabara, D., A. Ernest, and B. Mpondo, *Health facility service availability and readiness to provide basic emergency obstetric and newborn care in a low-resource setting: evidence from a Tanzania National Survey.* BMJ open, 2019. **9**(2): p. e020608.

5.	Lama, T.P., et al., *Assessment of the Quality of Antenatal Care, Family Planning, and Sick Child Care Services in Nepal: Further Analysis of the 2015 Nepal Health Facility Survey. DHS Further Analysis Reports No. 123*. 2019, ICF.

6.	Bintabara, D. and B.C.T. Mpondo, *Preparedness of lower-level health facilities and the associated factors for the outpatient primary care of hypertension: Evidence from Tanzanian national survey.* PloS one, 2018. **13**(2): p. e0192942.

7.	World Health, O., *Service availability and readiness assessment (SARA): an annual monitoring system for service delivery: reference manual*. 2014, World Health Organization.
