# Supplementary material for: Are Bangladeshi healthcare facilities prepared to provide antenatal care services? Evidence from two nationally representative surveys
Source: PLOS Glob Public Health. 2022 Jul 25;2(7):e0000164. doi: 10.1371/journal.pgph.0000164 (PMC10021659; doi:10.1371/journal.pgph.0000164)
Supplement: Attachment — Submitted filename: Authors Response_PGPH-D-21-00564R2_06-06-2022.docx [file pgph.0000164.s003.docx]

**Reviewer #1:** Thank you for addressing the comments and revising the strategy to be a 3-level indicator of readiness. I also appreciate the updated tables and supplementary table focusing on description of the health system. For Appendix Table 1, you could consider turning this into a histogram would be easier to interpret.

**Authors Response:** We thank the reviewer for the thoughtful comments and suggestions. We turned Appendix Table 1 into a histogram. Please see Appendix Figure 1.

**Reviewer #2:** I want to thank the authors for undertaking this substantial revision. I appreciate the additional detail on the study design and updating the main analyses from a binary to categorical outcome. However, I have new comments in response to the updated statistical analyses, in addition to prior comments that I do not believe were adequately addressed in the revision.

**Authors Response:** We appreciate the reviewer providing thoughtful feedback on the manuscript.

*New Comments*

I appreciate that the authors made the binary outcome with cut-off at 50% into a categorical variable. However, the cutoffs of < 62.5%, 62.5%-66.7%, >66.7% seems arbitrary. In the studies referenced (reference numbers 30-32), different cutoffs were used. For example, in references 30 and 32, <33.3%, 33.3%-66.7%, >66.7% were used as cutoffs. I realize that in this data, there are very few facilities < 33.3%, which is likely why the authors chose not to do this. **As a solution:** I suggest using a data-driven approach to choose category cutoffs. The authors can find the 33rd-percentile and 66th-percentile of the scores and use those as the cut-offs for three groups. Notably, I believe this will result in similar cut-offs to what the authors currently have.

**Authors Response:** As per your suggestion, we divided the ANC service preparedness score into three equal sections using the 33rd and 66th percentiles. Please see page 9-10. The results from this new categorization were slightly different from the previous one. We provided the new results (using the 33rd and 66th percentiles) in the manuscript.

The authors statement that: “We tried to use a linear regression model to identify the
significant potential factors by considering the outcome variable “ANC service preparedness” as a continuous variable. But the histogram of the outcome variable indicates that it does not follow a normal distribution (Appendix Figure 1)” is not valid as you can still perform linear regression if the outcome variable is non-normal. Normality of the outcome is not a necessary assumption. In addition, there is no Appendix Figure 1 – just Appendix Table 1. **As a solution**: I suggest that the authors remove this sentence and just describe the polytomous outcome / multinomial regression model, which is a valid approach. In addition, I would recommend replacing Appendix Table 1 with a histogram (create a figure).

**Authors Response:** We removed the sentences and just described the multinomial logistic regression model. Please see page 11. Moreover, we replaced appendix table 1 with a histogram (Appendix Figure1).

Please state which cutoffs were used for the basic amenities and infection prevention scores when defined in the “Potential factors” section.

**Authors Response**: We provided the cut-offs of the basic amenities and infection prevention score variables. Please see page 12.

For the multinomial regression, the coefficients correspond to a “relative risk ratio”, which compares risk of outcome by group, not the odds. However, the authors accidentally left the word “odds” in the Abstract and Results. Please update and verify wording is appropriate throughout.

**Authors Response:** We updated the abstract and removed the word “odds”.

*Prior comments that were not adequately addressed in the revision:*

In my major comments, I asked the authors to provide a more detailed discussion on the ANC preparedness index by either comparing the distribution of scores in Bangladesh to other countries or WHO.” The authors stated that this was done in the Discussion, but I think more nuance and detail is needed (including mention of the actual scores from other countries). **One statement I thought was especially confusing:** The authors stated that “the overall preparedness score for the ANC service was determined to be poor” but then classified Tanzania as having adequate birth preparedness based on a prior study. However, I think the birth preparedness raw scores were higher in Bangladesh than in both Tanzania (reference 30) and Haiti (reference 32). These two statements seem to be
at odds.

**Authors Response**: Quality and the readiness of ANC services are two distinct concepts. The health facility surveys of other low and middle-income countries like Tanzania, Haiti, Malawi, Nepal, etc. did not provide information on ANC readiness scores and gave information on measures of quality based on observations of ANC consultations as well as client exit interviews, but the Bangladesh Health Facility Survey only gives information on service availability and readiness. Therefore, we were unable to compare the ANC readiness score of Bangladesh with other countries. Moreover, we corrected the confusing statement.

The previous study (reference number 30 in the manuscript) looked at the current status of family planning services in Tanzania and the factors that affect clients' satisfaction. Also, the study (reference number 32 in the manuscript) aimed to explore the influence of service readiness on the use of facility delivery care. Both of these two studies did not measure the ANC preparedness score.

In response to one of my prior comments about non-response, the authors stated that “there were 15 and 14 missing values in the variable ‘Routing quality assurance activities’ in 2014 and 2017 survey respectively. The missing values were excluded in the regression modeling.” However, the exclusion of missing values is not mentioned in the text – please include in Data Analysis and/or Results section.

**Authors Response:** We included the exclusion of missing values in the data analysis section. Please see page 13.

In their response, the authors stated that they clarified the meaning of “availability of tracer indicators” in the main text, but I do not see where this has been updated. Please refer to the exact phrase.

**Authors Response:** We clarified the meaning of the availability of tracer indicators in the outcome variable and results sections. Please see page 9 and 13.

Instead of “The final study samples were drawn from all facilities that provide ANC services.”, I would phrase this as “Facilities that provided ANC services were included in the analysis, resulting in 1508 facilities in 2014 BHFS and 1506 facilities in 2016 BHFS.”

**Authors Response:** We rephrased it according to the suggestion of the reviewer.
